# Development and Validation of a Prediction Pneumothorax Model in CT-Guided Transthoracic Needle Biopsy for Solitary Pulmonary Nodule

**DOI:** 10.1155/2019/7857310

**Published:** 2019-05-05

**Authors:** Saibin Wang, Junwei Tu, Wei Chen

**Affiliations:** ^1^Department of Respiratory Medicine, Jinhua Municipal Central Hospital, Jinhua 321000, Zhejiang Province, China; ^2^Department of Radiology, The Second Affiliated Hospital and Yuying Children's Hospital of Wenzhou Medical University, Wenzhou 325027, Zhejiang Province, China

## Abstract

Computed tomography-guided transthoracic needle biopsy (CT-TNB) is widely used in the diagnosis of solitary pulmonary nodule (SPN). However, CT-TNB-induced pneumothorax occurs frequently. This study aimed to establish a predictive model for pneumothorax following CT-TNB for SPN. The prediction model was developed in a cohort that consisted of 311 patients with SPN who underwent CT-TNB. An independent external validation cohort contained 227 consecutive patients. The least absolute shrinkage and selection operator (Lasso) regression analysis was used for data dimension reduction and predictors selection. Multivariable logistic regression was used to develop the predictive model, which was presented with a nomogram. Area under the curve (AUC) was used to determine the discrimination of the proposed model. The calibration was used to test the goodness-of-fit of the model, and decision curve analysis (DCA) was used for evaluating its clinical usefulness. Five variables (age, diagnosis of nodule, puncture times, puncture distance, and puncture position) were filtered by Lasso regression. AUC of the predictive model and the validation were 0.801 (95% CI, 0.738-0.865) and 0.738 (95% CI, 0.656-0.820), respectively. The model was well-calibrated (P > 0.05), and DCA demonstrated its clinical usefulness. Thus, this predictive model might facilitate the individualized preoperative prediction of pneumothorax in CT-TNB for SPN.

## 1. Introduction

With the development of imaging technology and the improvement in health consciousness among people, the detection rate of asymptomatic solitary pulmonary nodule (SPN) has increased in the past few years [1]. Reportedly, the malignant rate of SPN ranges from 10% to 68% in clinical practice [2]. Therefore, pathological diagnosis of SPN is of utmost importance to avoid missing diagnosis of early lung cancer [3].

The Fleischner Society Guidelines were established and are continuously updated for the management of solitary pulmonary nodule (SPN) [4]. Radiographic follow-up is essential for the management of pulmonary micronodules. However, biopsy is often required for SPN larger than 1.0 cm in diameter. Due to its several advantages, computed tomography-guided percutaneous transthoracic needle biopsy (CT-TNB) is still a preferred biopsy method to obtain tissues for pathological examination to date [5]. However, it is noteworthy that, as a complication, CT-TNB-induced pneumothorax occurs quite commonly with a frequency of 24-60%, and the incidence of pneumothorax requiring chest tube placement ranges from 2.2% to 14.2% [6–11].

Currently, CT-TNB is commonly performed in an outpatient setting. The biggest complication in outpatient management is not the occurrence of the pneumothorax per se, but an increase in pneumothorax occurrence that requires chest tube drainage and patient hospitalization [12, 13]. Accurate prediction of postoperative pneumothorax will help clinicians to screen the high-risk population undergoing CT-TNB and thus further contribute to perioperative management of these patients, such as preoperative preparation (e.g., using oxygen-absorption mask), intraoperative procedures (e.g., reducing biopsies), and postoperative follow-up (e.g., extending observation period). Therefore, it is crucial to accurately predict the occurrence of pneumothorax after CT-TNB. The purpose of this study was to establish a risk prediction model for pneumothorax in CT-TNB for SPN.

## 2. Materials and Methods

### 2.1. Study Design and Ethics Statement

A total of 311 patients identified as SPN who underwent CT-TNB at a tertiary hospital (Jinling Hospital of Nanjing University, Nanjing, China) between January 2004 and January 2014 were used to develop a clinical prediction model [3]. An independent external validation cohort of 227 consecutive patients was enrolled at another tertiary hospital (Jinhua Hospital of Zhejiang University, Jinhua, China) from June 2016 to December 2017. The diameters of all biopsy nodules, measured using lung window on CT image, were between 7.5 mm and 29.5 mm. The study was approved by the ethics committee of Jinhua Hospital of Zhejiang University and conducted according to Declaration of Helsinki guidelines. All patient information was handled anonymously, and informed consent was, therefore, waived.

### 2.2. CT-TNB Procedure and Data Collection

Patients underwent CT-TNB in different positions (prone, supine, or lateral position) based on the shortest distance from the lesion to the body surface. All biopsies were executed by three clinicians experienced in pneumology and radiology in the derivation cohort and by one clinician in the validation cohort. A coaxial 18-gauge needle (REXK0682; Bard Peripheral Vascular, Inc., Tempe, AZ) was used in all biopsies. Generally, one biopsy was performed, and occasionally a second or third biopsy was performed when a previous biopsy had failed. In this study, the puncture distance was defined as the length between the pleura and the center of the SPN.

After CT-TNB, patients maintained supine for at least 6 hours and got out of bed 24 hours later. Postbiopsy pneumothorax was evaluated by CT scan either immediately after removal of the biopsy needle or during follow-up on demand. Chest tube placement was considered when a large pneumothorax (>30%) was found or when the patient developed significant symptoms associated with pneumothorax.

The following data was extracted from each patient: age, gender, the diagnosis of SPN (benign or malignant), location of SPN (periphery or nonperiphery), nodule size, puncture position, puncture times, puncture distance, biopsy pneumothorax (yes or no), and chest tube placement (yes or no).

### 2.3. Statistical Analysis

Descriptive statistics were used to summarize baseline characteristics. Continuous variables were presented as median (25%-75% interquartile) and categorical variables were expressed as the number (percentage). Between-group comparisons were performed using unpaired t-tests (normal distribution) or Kruskal-Wallis rank sum test (nonnormal distribution), Pearson chi-squared tests, or Fisher's exact test, as appropriate. In this study, the least absolute shrinkage and selection operator (Lasso) regression method was used to determine the most useful predictors from the derivation dataset. Pneumothorax risk prediction model was created using logistic regression and was presented with a nomogram. Area under the receiver operating characteristic curve (AUROC) was used to determine the discrimination of the predictive model; calibration curves were plotted to assess the model accompanied with the Hosmer-Lemeshow test, and decision curve analysis (DCA) was performed to determine the clinical usefulness of the model by quantifying the net benefits at different threshold probabilities in the validation dataset [14]. Statistical analyses were performed using R software (version 3.5.1; http://www.r-project.org). P < 0.05 was considered statistically significant.

## 3. Results

### 3.1. Clinical Characteristics

In the derivation cohort, 17.7% (95% confidence interval [CI], 13.4-21.9%) had pneumothorax after CT-TNB, with 3 cases (5.5% of the pneumothorax) requiring chest tube placement, while in the validation cohort, the incidence of pneumothorax was 22.5% (95% CI, 17.0-27.9%), with 1 case (2.0% of the pneumothorax) requiring chest tube drainage. Patient's demographics and puncture features are shown in Table 1.

### 3.2. Predictors Entering the Model

**Figure 1:**
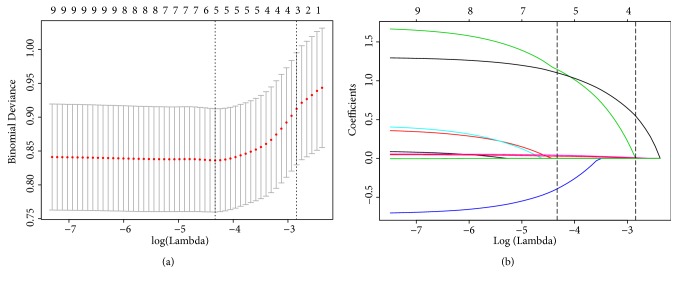
Predictors' selection using the Lasso regression method. (a) A 10-fold cross-validation was used in the Lasso regression. Binomial deviance was plotted versus log (lambda). Dotted vertical lines were drawn at the optimal values by utilizing the minimum criteria (left dotted line) and the 1 standard error criterion (right dotted line). (b) Lasso coefficient profiles of the 8 variables. A coefficient profile plot was produced against the log (lambda) sequence. Dotted vertical lines were drawn at the optimal values by using the minimum criteria (left dotted line) and the 1 standard error criterion (right dotted line). In the current study, predictors were chosen according to the minimum criteria, where optimal lambda resulted in 5 nonzero coefficients.

Of variables, 8 were reduced to 5 potential predictors on the basis of 311 patients in the derivation cohort and were features with nonzero coefficients in the Lasso regression analysis (Figure 1). These variables were age (coefficient, 0.043), the diagnosis of SPN (coefficient, -0.391), puncture times (coefficient, 1.103), puncture distance (coefficient, 0.030), and puncture position (coefficient, 1.146).

### 3.3. Discriminative Power of the Model and Nomogram

**Figure 2:**
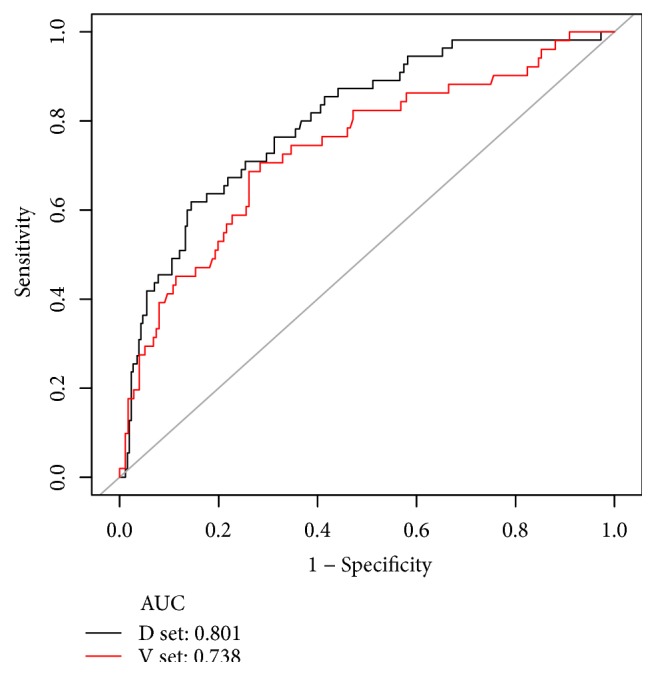
ROC curves of the predictive model in the derivation cohort and the external validation cohort. Area under the ROC curve (black line) shows the predictive ability of the model in the derivation cohort, and area under the ROC curve (red line) validates the predictive ability of the model. ROC, receiver operating characteristic.

**Figure 3:**
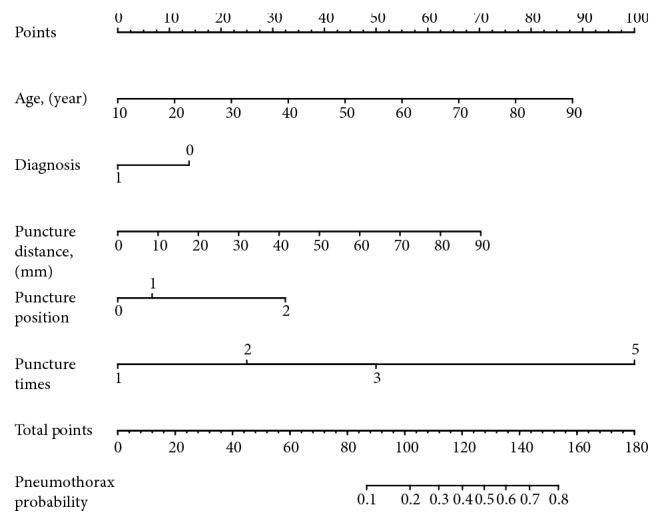
Nomogram for prediction of post-CT-TNB pneumothorax risk and its predictive performance. First of all, find point for each predictor (variable) of a patient on the uppermost rule; then add all points together and calculate the “total points”; finally find the corresponding predicted probability of pneumothorax on the lowest rule. Codes annotation: diagnosis, 0: benign, 1: malignant; puncture position, 0: supine, 1: prone, 2: lateral position. CT-TNB, computed tomography-guided percutaneous transthoracic needle biopsy.

A risk prediction model for SPN in CT-TNB-induced pneumothorax was established based on the aforementioned 5 risk predictors. As shown in Figure 2, the AUC of the predictive model (black line) was 0.801 (95% CI, 0.738-0.865). The AUC of the external validation (red line) was 0.738 (95% CI, 0.656-0.820). To provide physicians with a quantitative tool to predict individual probability of pneumothorax post-CT-TNB, a nomogram was established based on multivariable logistic analysis in the derivation cohort (Figure 3).

### 3.4. Calibration of the Model

**Figure 4:**
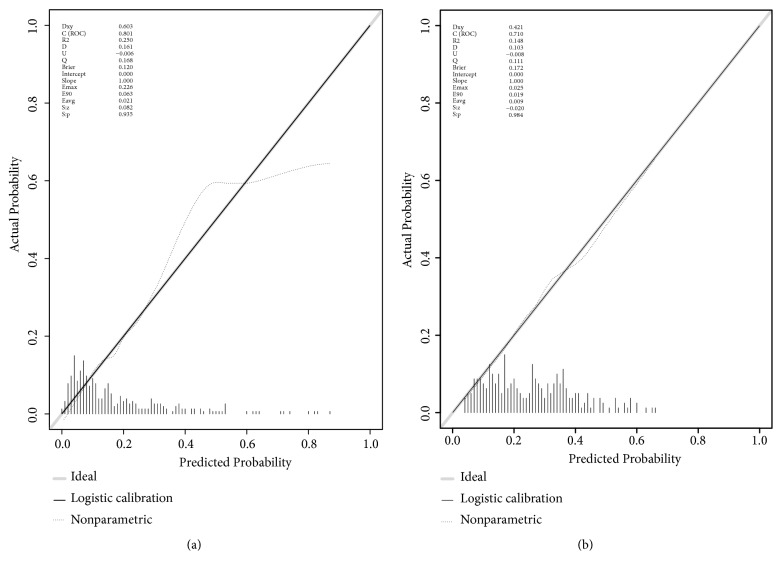
Calibration curves of the model and the validation. (a) Calibration curve of the model in the derivation cohort (Emax = 0.226, Eavg =0.021, P = 0.935). (b) Calibration curve of the model in the validation cohort (Emax = 0.025, Eavg =0.009, P = 0.984). Calibration curve shows the calibration of the model in terms of the agreement between the predicted risks of pneumothorax post-CT-TNB and observed outcomes of pneumothorax post-CT-TNB. The y-axis represents the actual pneumothorax rate post-CT-TNB. The x-axis represents the predicted risk of pneumothorax post-CT-TNB. The shadow line represents a perfect prediction by an ideal model. The dotted line represents the performance of the model, of which a closer fit to the shadow line represents a better prediction.

The calibration curve of the model for the probability of pneumothorax post-CT-TNB demonstrated good agreement between prediction and observation in the derivation cohort (Figure 4(a)). The Hosmer-Lemeshow test yielded a nonsignificant statistic (P = 0.935), which suggested that there was no departure from perfect fit. Similarly, there was goodness-of-fit in the validation cohort (Figure 4(b), P = 0.984).

### 3.5. Decision Curve Analysis of the Model

**Figure 5:**
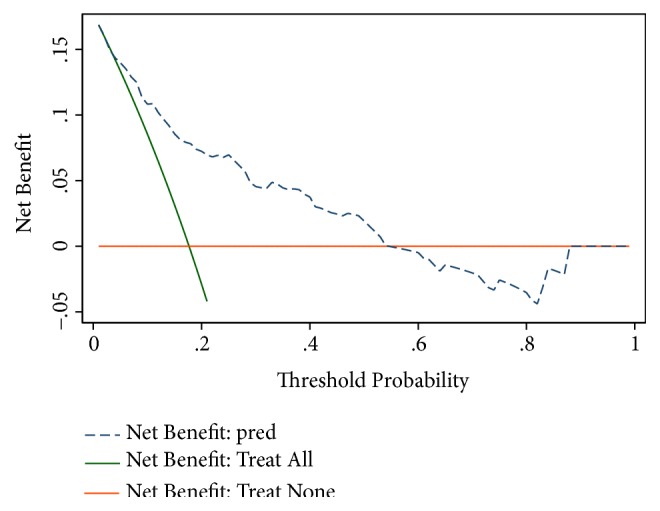
Decision curve analysis for the predictive model and the validation. The y-axis measures the net benefit. The dotted line represents the model. The black line represents the assumption that all patients have pneumothorax post-CT-TNB. Thin orange line represents the assumption that no patients have pneumothorax post-CT-TNB. The decision curve shows that if the threshold probability of an individual is < 55%, using this model to predict pneumothorax post-CT-TNB adds more benefit than either the treat-all tactics or the treat-none tactics.

The DCA for the model is presented in Figure 5. The decision curve revealed that if the threshold probability of an individual was < 55%, using this model to predict post-CT-TNB pneumothorax adds more benefit than either the treat-all tactics or the treat-none tactics.

## 4. Discussion

In the present study, a risk prediction model for pneumothorax was established in patients undergoing SPN CT-TNB. This prediction model incorporates five items of age, the diagnosis of SPN, puncture times, puncture distance, and puncture position. The model has good predictive ability both in the derivation cohort (AUC: 0.801) and in the external validation cohort (AUC: 0.738). In addition, a nomogram was constructed based on the aforementioned predictors which facilitate individualized prediction of pneumothorax post-CT-TNB.

SPN is a mass in the lung smaller than 3.0 cm in diameter and typically presented as a single, discrete, round, or oval opacity lesion [15]. The possibility of cancer in solitary SPN ranges from 10% to 68% in clinical practice [2]. In the last few decades, the detection rate of SPN has increased, with a reported detection rate between 8% and 51% [1]. To get a defined diagnosis of SPN, ultrasound, CT, electromagnetic navigation bronchoscopy, and endobronchial ultrasonography are utilized for imaging guidance in lesion biopsy. Among these, CT-TNB is still the most frequently used modality in clinical practice [5]. Common complications of CT-TNB include pneumothorax, hemoptysis, hemothorax, air embolism, and infection, with pneumothorax being the most frequent complication. The incidence of pneumothorax has been reported to be from 24 to 60% and the incidence of pneumothorax requiring chest tube placement ranges from 2.2% to 14.2% [6–11]. The risk of this complication in outpatients would be more significant [5]. It is critical to accurately predict the probability of a pneumothorax after CT-TNB. Only a few predictive models are available for this. Zhao et al. created a predictive model that obtained 0.735 AUC of ROC in a study cohort with 864 cases. The study however did not consider the size of lesions, and the model has not been validated in their study [16]. In the present study, a risk prediction model was established based on 5 predictors proposed by shrinking the regression coefficients with the Lasso regression. Reportedly, this method has surpassed the method of choosing predictors based on the strength of their univariable association with outcome [17]. All these variables are easily available clinically. To the best of our knowledge, a predictive model of pneumothorax risk in patients with SPN undergoing CT-TNB has not been previously reported. This prediction model is both of good discrimination and calibration.

In addition, to justify the clinical usefulness, DCA was used to assess whether the decisions on the basis of this prediction model would improve patient outcomes. This novel method is based on the threshold probability to gain insight into the clinical consequences and to weigh the net benefit [14, 18, 19]. The decision curve showed that if the threshold probability of an individual is < 55%, using the model in the present study to predict pneumothorax post-CT-TNB adds more benefit than either the treat-all tactics or the treat-none tactics. The proposed model would be useful in the management of patients with SPN undergoing CT-TNB. It will help clinicians identify patients who are at a high risk of postoperative pneumothorax, then adjust preoperative preparation for these patients (e.g., using oxygen-absorption mask), and improve intraoperative procedure (e.g., reducing biopsies and/or applying a finer biopsy needle) and postoperative management (e.g., extending observation period). This may further contribute to reducing the incidence of pneumothorax and enabling patients to receive timely treatment, especially for patients who need chest tube drainage. Some limitations of this study are worth noting. First, although an independent external validation of the model was carried out, more verification in multiple centers is still needed to confirm the utility of the predictive model. Second, some risk factors, like emphysema, were not included in this study because this prediction model was built based on public data [20].

## 5. Conclusion

This predictive model can be used to identify SPN patients with higher risk of pneumothorax undergoing CT-TNB and, when possible, to implement primary prevention strategies such as reducing the number of pleural punctures, oxygen administration, and higher postoperative observation and evaluation of pneumothorax.

## Figures and Tables

**Table 1:** Clinical characteristics of the derivation cohort and the external validation cohort.

Variables	Pneumothorax in the derivation cohort		P value	Pneumothorax in the validation cohort		P value
	No (n=256)	Yes (n=55)		No (n=176)	Yes (n=51)	
Sex, n (%)			0.393			0.881
Female	85 (33.20)	15 (27.27)		78 (44.32)	22 (43.14)	
Male	171 (66.80)	40 (72.73)		98 (55.68)	29 (56.86)	
Age, (years)	59 (51-67)	67 (60-72)	<0.001	61 (51-68)	63 (58-73)	0.016
Diagnosis of nodule, n (%)			0.552			0.314
Benign	69 (26.95)	17 (30.91)		86 (48.86)	29 (56.86)	
Malignant	187 (73.05)	38 (69.09)		90 (51.14)	22 (43.14)	
Nodule location, n (%)			0.495			0.776
Non-periphery	72 (28.12)	18 (32.73)		27 (15.34)	7 (13.73)	
Periphery	184 (71.88)	37 (67.27)		149 (84.66)	44 (86.27)	
Puncture position, (%)			0.022			<0.001
Supine	109 (42.58)	20 (36.36)		71 (40.34)	5 (9.80)	
Prone	130 (50.78)	25 (45.45)		74 (42.05)	37 (72.55)	
Lateral	17 ( 6.64)	10 (18.18)		31 (17.61)	9 (17.65)	
Nodule size, (mm)	20.25 (17.50-24.50)	20.50 (18.00-24.25)	0.970	20.00 (15.00-25.00)	19.00 (15.00-25.00)	0.607
Puncture distance, (mm)	26.5 (19.0-40.0)	32.0 (24.5-48.5)	0.002	15.0 (5.0-21.0)	12.0 (5.0-20.0)	0.374
Puncture times	1 (1-1)	1 (1-2)	<0.001	1 (1-2)	2 (1-2)	0.002

## Data Availability

The data used to support the findings of this study are available from the corresponding author upon request.
